# Efficacy of Digital Interventions for Anxiety Disorders: A Systematic Review and Meta-Analysis

**DOI:** 10.1192/j.eurpsy.2024.774

**Published:** 2024-08-27

**Authors:** H.-G. Ji, S. Kang, H. Bae, G. Kim, J.-W. Hur

**Affiliations:** ^1^Department of Psychology, Korea University, Seoul, Korea, Republic Of

## Abstract

**Introduction:**

Anxiety disorders are one of the most common mental disorders, yet only less than 20% of people with anxiety disorders receive adequate treatment. Digital interventions for anxiety disorders can potentially increase access to evidence-based treatment. However, there is no comprehensive meta-analysis study that covers all modalities of digital interventions and all anxiety disorders.

**Objectives:**

A preliminary meta-analysis was conducted to examine the treatment efficacy of digital interventions [e.g., virtual reality (VR)-, mobile application-, internet-based interventions] for anxiety disorders and to identify potential moderators that may lead to better treatment outcomes.

**Methods:**

We searched Embase, PubMed, PsycINFO, Web of Science, and the Cochrane Library for randomized controlled trials examining the therapeutic efficacy of digital interventions for individuals with anxiety disorders from database inception to April 18, 2023. Search keywords were developed by combining the PICOS framework and MeSH terms. Data screening and extraction adhered to PRISMA guidelines. We used a random-effects model with effect sizes expressed as Hedge’s *g.* The quality of the studies was assessed using the Revised Cochrane risk-of-bias tool for randomized trials (RoB 2). The study protocol was registered in PROSPERO on April 22, 2023 (CRD42023412139).

**Results:**

A systematic literature search identified 19 studies with randomized controlled trials (21 comparisons; 1936 participants) with high overall heterogeneity (*Q* = 104.49; *P* < .001; *I*^2^ = 80.9%). Digital interventions reduced anxiety symptoms with medium to large effect sizes ( *g* = 0.78; 95% CI: 0.55-1.02; *P* < .001), with interventions for specific phobia showing the largest effect size (*n* = 6; *g* = 1.22; 95% CI: 0.51-1.93; *P* < .001). VR-based interventions had a larger effect size (*n* = 6; *g* = 0.98; 95% CI: 0.39-1.57; *P* < .001) than mobile- or internet-based interventions, which had medium effect sizes. Meta-regression results exhibited that effect sizes of digital interventions were associated with the mean age of participants (*β* = 0.04; 95% CI: 0.02-0.06; *P* < .001).

**Conclusions:**

The results of this study provide evidence for the efficacy of digital interventions for anxiety disorders. However, this also suggests that the degrees of effectiveness in reducing anxiety symptoms can be moderated by the specific diagnosis, the modalities of digital technologies, and mean age, implying that the application of digital interventions for anxiety disorders should be accompanied by personalized guidance.

**Disclosure of Interest:**

None Declared

